# Is Campus a Place of (In)Security and Crime? Perceptions and Predictors among Higher Education Students

**DOI:** 10.3390/ejihpe12020015

**Published:** 2022-02-02

**Authors:** Vanessa Azevedo, Laura M. Nunes, Ana Sani

**Affiliations:** 1 Faculty of Human and Social Sciences, University Fernando Pessoa (UFP), 4249-004 Porto, Portugal; lnunes@ufp.edu.pt (L.M.N.); anasani@ufp.edu.pt (A.S.); 2 Observatory Permanent Violence and Crime (OPVC), University Fernando Pessoa (UFP), 4249-004 Porto, Portugal; 3 Centro de Investigação em Justiça e Governação, University of Minho (UM), 4710-057 Braga, Portugal; 4 Research Center on Child Studies (CIEC), University of Minho (UM), 4710-057 Braga, Portugal

**Keywords:** violence, college, university, criminality, diagnosis of local security, subjective insecurity

## Abstract

This paper addresses subjective insecurity, namely perceptions of (in)security and criminal variables on campus among Portuguese higher education students. Additionally, predictors of perceptions of (in)security and gender differences were also examined. The participants were 775 students and data were collected through the “Diagnosis of Local Security Questionnaire”. Robbery, physical assault, theft, and sexual offenses were the most feared crimes. Additionally, robbery, theft, and public property damage were perceived as the most common on campus. Alcohol/drug consumption and juvenile conflicts/delinquency were the main reasons justifying criminal occurrences. Sociodemographic variables such as gender, age, education, and years of campus attendance, as well as criminal variables (e.g., perceived trend of crime, criminal occurrences, and crime promoters) predicted perceptions of (in)security. Females reported more fear than males of robbery, sexual offenses, physical aggression, and domestic violence. Therefore, preventive measures, including in the social domain and physical spaces, are mandatory to reduce violence on campus.

## 1. Introduction

Security and education are part of the Universal Declaration of Human Rights and we expect that educational institutions constitute safe places. Higher education campuses are among those and had traditionally been seen as protected from the criminality and danger that affected outside communities [1,2]. Nonetheless, especially in USA, there is increasing awareness about safety on campuses and the topic is widely recognized as a political and social issue [2,3,4]. Consequently, the topic has, on the one hand, been extensively addressed by researchers and, on the other, been the focus of administrators, faculty/staff, and police forces, which implemented a diversity of practical measures, as we can see in [5].

Previously to the theoretical overview, some conceptual issues about (in)security should be enlightened. From a common-sense point of view, according to the Portuguese Dictionary [6], security (the Portuguese word is “segurança”) evolved from the Latin secūrus, related to the absence of worries/concerns. According to the online version of the Cambridge Dictionary [7], safety means “a state of being safe from harm or danger”, being applied to different settings (i.e., road safety). When applied to the criminological field, the concept includes not only people but also their belongings. Additionally, it also covers formal and informal social control, strategies and political measures for criminal prevention, and perceptions of (in)security [8]. Therefore, authors such as Zedner (2009) have claimed that security is a broad and comprehensive concept [9]. The current study addressed perceptions of (in)security, which, based on the work of Guedes as cited in [8], can be defined in two distinct dimensions: objective and subjective. The dimension of objective insecurity includes factual information about juvenile delinquency, urban disorders and incivilities, and predatory crimes, being collected through official statistics or self-report surveys. Moreover, objective insecurity impacts on subjective insecurity. This subjective dimension includes fear of crime, risk perception, and safety behaviors. Fear of crime and risk perception should be conceptually distinguished: namely, while the first consists of an emotional reaction toward the self or others, risk perception addresses a cognitive dimension. In a previous study [10], we found relevant results for objective insecurity: for instance, around 9% of the students were direct victims, and nearly 40% were indirect victims. Moreover, males presented a high risk for direct victimization, but there were no gender differences on indirect victimization. The remaining question is: what about subjective insecurity?

### 1.1. Previous Research

A couple of empirical studies have focused on the perception of (in)security by higher education students [11,12]. For instance, Jennings, Gover, and Pudrzynska assessed security perception using a five-point Likert scale (ranging from 1 to 5) and found a mean value of 3.87 [13]. Additionally, students were also asked about fear of crime (*M* = 2.58) and perceived risk (*M* = 3.39). More recently, Merianos, King, and Vidourek asked students how safe did they feel, and they found that the majority of students answered positively [14]. Wilcox, Jordan, and Pritchard concluded that 15.5% of the college students felt unsafe on campus [15]. In more detail, they were particularly worried about sexual violence (41.87%), about physical violence (38.42%), and about stalking (21.83%)—all crimes being perpetrated by strangers. In other study, about property crime [11], feared crimes varied by the time of occurrence: indeed, during the day, the most feared crimes were burglary (21%), vandalism (17%), and property theft and vehicle theft (16%); at night, students most feared burglary while away (30%), vehicle burglary (26%), and vehicle theft (25%).

Gender differences have a long tradition in criminological research, including on higher education campuses [16,17], exhibiting a phenomenon labelled as the fear–gender paradox. Indeed, empirical evidence suggests that: (i) victims are mainly males [18]; (ii) females and males tend to be victims of different crimes (i.e., males reported mainly theft and physical assault, while females presented high rates of sexual assault) [1]; and (iii) females tend to exhibit higher values of fear of crime than males [19]. Findings that females are more fearful of crime [20] are explained as related to female vulnerability. The authors of [21] argued that gender differences are socially constructed, although research seems to be gender biased, stressing the female point of view. Specific frameworks [22,23], analysis of female-only samples, applied questions, as well as traditional explanatory reasons can be viewed as evidence of this female predominance. To overcome these limitations, Reid and Konrad developed a study to compare crimes that are gender neutral to those that disproportionately target males (i.e., robbery) or females (i.e., sexual assault) and found that there were no differences on the gender-neutral crimes (i.e., burglary), while females reported statistically high values of fear of crime on both sexual assault and robbery [21]. In an effort to further clarify the robbery result, the authors analyzed the interaction between perceived risk and gender. They noted that fear of robbery remained stable in females independently from perceived risk, while in males the increase in perceived risk seemed to be related to higher values of fear of robbery. Applying an alternative approach, Sutton and Farrall studied fear of crime and social desirability and found that females presented high levels of fear of burglary, vandalism, assault, and overall crime; they also tended to score high on the lie scale [24]. When the authors analyzed all the participants and a female-only subsample, there were no correlations between social desirability and fear of crime. In males, there was a significant negative correlation relating to burglary, vandalism, and total crime, suggesting that those that reported higher values on the lie scale tended to report low values of fear of crime. Moreover, after the removal of the estimated effect of socially desirable responding, the researchers found that the mean values of fear of crime were higher for males than for females.

### 1.2. Theoretical Background

Empirical data suggest that campuses are safer than the community in which they are located [25]. However, according to situational opportunity theories—for a recent review see Wilcox [26]—namely, the lifestyle–routine activities theory [27] and situational crime prevention through environmental design (CPTED) [28], campuses can be a hotspot for crime, and college students may be a risk group [29]. According to the lifestyle–routine activities theory, risk is a product of the interaction between time and space and the presence of some factors: motivated offenders, target and victim’s proximity, target suitability, and lack of adequate guardianship. Briefly, the CPTED theory proposes that crime opportunities may be decreased through urban building and the concept of defensible space [28]. CPTED focuses on design through seven key strategies: territorial reinforcement, surveillance, image, access control, legitimate activity support, target hardening, and the surrounding environment [30]. For instance, in a recent work by Maier and DePrince, it was found that a statistically significant relationship between perceptions of safety and a university’s efforts to increase the perception of security and reduce fear, including increased security patrols, ID access to buildings, adequate lighting, and campus safety services [31]. Similarly, Chekwa, Thomas, and Jones asked students “what should be done to make your campus safer?” and concluded the most important measures were adding security officers, cameras, emergency call boxes, and improving lighting [32].

A campus is a free public space attended by a large transient population, which concentrates a high-offending group, namely young males [33]. Moreover, students seem to represent a specific group: they seem to perceive themselves as largely invulnerable to risk, engaging in few protective measures, and seem to be prone to alcohol and recreational drug consumption, while, concomitantly, they own a high number of expensive belongings (e.g., laptops, watches, mobile phones), and usually they are away from household members and family [29,34]. Attending to these risk factors, authors Cozens and Love [30] claimed that “students and the general public have different experiences” (p.153).

### 1.3. Current Study

Despite the international context, the USA especially has remained particularly worried about (in)security and crime perceptions on university campuses [2,35] have claimed that it is demanding to study different cultures. In Portugal, these topics remained neglected for decades, and to our best knowledge—besides our research—there is only a single study about campus victimization, analyzing a different campus [36] and focusing only on objective insecurity. Currently, Portuguese institutions have not developed specific legislation nor reporting practices, and there are no specific resources/programs or security policies to address campus crime. Contrastingly, the media have recently been paying increased attention to the topic, especially to violent episodes [37,38], similarly to the international scenario [39,40].

Porto’s campus is openly located at the urban parish of Paranhos and comprises both public and private colleges/universities covering different scientific domains. Therefore, the campus is attended by a large and heterogeneous population, not restricted to students nor university staff, but also including inhabitants and passersby. According to official data, Porto is the second Portuguese city to present the highest values on crime [41]. At this point, we cannot be sure if the campus is actually safer than the local community where it resides or not, making it urgent to collect data about the topic. In a previous study [10], we found that the majority of students felt safe in Asprela’s area (73.4%, *n* = 569). However, we did not explore explanatory reasons nor the predictors of perception of (in)security based on sociodemographic and criminal variables.

The current study aims to fill a gap in the previous research by examining variables that to our best knowledge have not been previously studied and by presenting Portuguese data (i.e., from Porto’s campuses) about two general topics, namely: (i) perceptions of (in)security and crime, (ii) sociodemographic and criminal predictors of perception of insecurity, and (iii) gender differences on these variables. More specifically, the first aim included a descriptive approach about (a) explanatory reasons for the perception of (in)security, (b) the most feared crimes, (c) the perception of occurrence (including specific personal and property crimes), (d) the perceived trend of crime and its explanatory reasons, and (e) factors that promote crime. Additionally, an inferential approach was performed to identify predictors of perception of (in)security based on sociodemographic and criminal variables. Our third aim was to compare females and males regarding: (a) most feared crimes, (b) the perception of occurrence, (c) perceived trend of crime, and (d) factors that promote crime.

## 2. Materials and Methods

### 2.1. Participants

It being unpractical to collect a representative sample, a convenience sample was instead studied. Participants were recruited among individuals studying at Porto’s campuses, which includes universities, colleges, and schools, the majority being public institutions (78.57%) and 21.43% being private. A total of 780 students were enrolled in this study; four cases were removed from the sample data for duplicated information or non-response. 

Table 1 presents a description of the final sample, which was composed of 775 students, 54.1% (*n* = 419) female, with a mean age of 21.76 years (*SD* = 5.11, ranging from 17 to 56). More than 90% of the participants were Portuguese (94.3%, *n* = 731) and single (93.8%, *n* = 727). Nearly 85% (*n* = 658) of the sample represented full-time students, and a similar percentage corresponded to undergraduate students (81.2%, *n* = 629). Moreover, 76.5% (*n* = 593) of individuals had attended the campus for 3 years or less, and 19.5% (*n* = 151) ranged from 4 to 6 years. Lastly, students tended to attend mixed universities (45.4%, *n* = 352), followed by Engineering schools (40.8%, *n* = 316).

### 2.2. Measures

Data were collected through the “Diagnosis of Local Security Questionnaire” [42], a self-report measure that had been specifically developed in collaboration with the Porto Metropolitan Police to evaluate objective and subjective features of (in)security (available as Supplementary material). It has been used intensively in different groups to perform local security audits [43,44,45].

The questionnaire is comprised of 136 closed or open-ended questions, allowing us to collect both quantitative and qualitative information. Questions were organized in five sections, namely, sociodemographic information, perception of (in)security, direct and indirect victimization, social control, and community participation. The current study focused only on sociodemographic variables and perception of (in)security. More specifically, besides sociodemographic information, participants were asked if they felt safe on campus (perception of (in)security, i.e., Do you consider that your study area is safe? Yes vs. No vs. Not answer/Did not know) and participants were further requested to explain their answers. Then, participants were asked to select those crimes that they thought of as frequent on Porto’s campus (perception of occurrence) and those they most feared from a list of fourteen crimes (e.g., fraud, robbery, sexual offense, domestic violence). Participants were asked if they thought that criminality had been increasing on campus (perceived trend of crime) and to provide explanatory reasons. Lastly, participants were asked to select from a list of twelve conditions those that promote crime occurrences (e.g., poverty/unemployment, poor lighting).

### 2.3. Procedures

After receiving the approval of the Internal Review Board, authorization for data collection was asked for from all the universities/schools included. Next, participants were invited to collaborate in a study about perceptions of (in)security and crime in the area where they studied, i.e., Porto’s campus. We presented the study procedures and conditions of participation to all individuals, and those that agreed to participate signed a written informed consent form. Self-reports surveys were gathered using a paper-and-pencil (collected in the institution’s surroundings and during classes) or online questionnaire (disseminated through institutional e-mails), according to the strategy of data collection established by each university/school. The measure took 20–30 min to be filled out. Participants did not receive any incentive to be enrolled in the study, participation being totally voluntary.

### 2.4. Statistical Analysis

The quantitative data were analyzed through the software IBM Statistical Package for Social Sciences (IBM SPSS for Windows, version 25.0, IBM Corp., Armonk, NY, USA). Being an exploratory, descriptive, and transversal study and attending to our aims, univariate descriptive statistics (i.e., frequencies, means, and standard deviations) were computed for all variables. Qualitative data (e.g., explanatory reasons) were first coded through thematic analysis and then further analyzed quantitatively. Additionally, inferential statistics were also performed, namely to identify predictors of perception of (in)security. We performed a binary logistic regression, and to compare groups we computed independent *t*-tests. The binary logistic regression, applying the Enter method, included two steps: first, only sociodemographic variables were analyzed, and the criminal variables were added in the second step.

## 3. Results

### 3.1. Perceptions about Insecurity and Crime

Those that felt safe explained it as due to previous experience/observation (64.5%, *n* = 367), formal social control (12.0%, *n* = 68), informal social control (10.2%, *n* = 58), by comparison to other areas (8.3%, *n* = 43), and other reasons (1.3%, *n* = 7). Oppositely, those that reported feeling unsafe (26.6%, *n* = 206) explained their answers based on the presence of crime/danger (48.1%, *n* = 99), urban environmental degradation/space distribution (16.0%, *n* = 33), previous experience/observation (12.1%, *n* = 25), difficulties in policing (11.2%, *n* = 23), occurrences during the night (8.3%, *n* = 17), associations with drugs consumption/traffic (1.0%, *n* = 2), and other reasons (2.4%, *n* = 5). As shown in Table 2, participants reported that robbery (67.9%, *n* = 526), physical assault (55.0%, *n* = 426), theft (44.4%, *n* = 344), and sexual offenses (31.6%, *n* = 245) were the most feared crimes. Oppositely, commercial property burglary (4.6%, *n* = 36) and domestic violence (including against children, intimates, and the elderly; range from 7.2–7.7%, *n* = 56–60) were perceived as less feared. The mean number of crimes feared by the participants was 3.12 (*SD* = 2.70, *range* = 0–13). Regarding perception of occurrence on campus, as presented in Table 1, robbery (43.9%, *n* = 340), theft (40.9%, *n* = 317), public property damage (39.0%, *n* = 302), and physical assaults (29.3%, *n* = 226) were the most reported. Domestic violence (including against children, intimates, and the elderly; range from 2.1–6.6%, *n* = 16–51) and arms traffic (1.2%, *n* = 9) were presented as less frequent. The mean number of crimes was 2.50 (*SD* = 2.17, *range* = 0–13).

Regarding the perceived trend of crime, the majority of the participants (72.5%, *n* = 562) did not think that crime was increasing. This perception was supported by previous experience/observation (70.1%, *n* = 394) and formal and informal social control, respectively 5.9% (*n* = 33) and 2.7% (*n* = 15). Occurrence of thefts and robberies (42.2%, *n* = 89), a problematic zone (18.5%, *n* = 39), economical deprivation/unemployment (11.8%, *n* = 25), previous experience/observation (11.8%, *n* = 25), mediatization (6.2%, *n* = 13), inefficient policing/laws (4.3%, *n* = 9), and drugs consumption/traffic (1.4%, *n* = 3) were the reasons pointed out by the participants that considered that crime was increasing (27.3%, *n* = 211). 

Participants identified, on average, 3.72 factors as crime promoters (*SD* = 2.32), especially alcohol/drug consumption (53.2%, *n* = 412), juvenile conflicts and delinquency (48.8%, *n* = 378), poverty/unemployment (46.1%, *n* = 357), insufficient policing (42.3%, *n* = 328), reduced presence of people during night (39.7%, *n* = 308), poor lighting (27.4%, *n* = 212), presence of strangers (27.0%, *n* = 209), and the low severity of punishment (24.3%, *n* = 188). A minority of participants also reported other factors, such as the incapacity to act on the part of police officers (19.1%, *n* = 148), family problems (16.1%, *n* = 125), poor accessibilities (14.6%, *n* = 113), or a lack of green spaces (6.5%, *n* = 50). Notwithstanding this, 6.7% (*n* = 52) of the participants did not answer nor present any factor.

### 3.2. Predicting Perception of (In)Security

A two-step binary regression model was tested to predict perceptions of insecurity among higher education students. To guarantee an outlier-free sample, only 694 participants were further analyzed. All the requirements related to binary regression were tested and fulfilled. In the first step, which included only sociodemographic variables, the model achieved statistical significance (*Χ*^2^(9) = 79.79, *p* < 0.001) and correctly classified 81.1% of the cases. As can be seen in Table 3, gender, age, years of attendance, and university domain were significant predictors. In the second step, after controlling for sociodemographic variables, the model also achieved statistical significance (*Χ*^2^(13) = 397.52, *p* < 0.001) and correctly classified 88.9% of the cases. All the criminal variables, except total of feared crimes, were individual predictors of perception of insecurity. In detail and according to the second step results, those that felt unsafe tended to be females, older, attending a postgraduate degree, and studying at the campus for four or more years. Moreover, those that perceived that criminality was increasing, that reported a high number of criminal occurrences, and that identified a high number of crime promoters seemed to be more unsafe.

### 3.3. Gender and Perceptions about Crime

Regarding perception of occurrence, 16.2% of females considered that crime was increasing while 11.1% of males reported the same perception; there was no association between gender and perception of occurrence, *Χ*^2^(1) = 2.97, *p* = 0.085.

Table 4 presents the descriptive data and chi-square tests for the most feared crimes, perception of occurrence, and factors that promote crime, comparing males and females. Regarding the most feared crimes, there were six significant associations; more specifically, females reported more fear than males of robbery, sexual offenses, physical aggression, and domestic violence (against children and intimate partners). Oppositely, theft was especially considered a feared crime by males. Moreover, there were gender differences on the total number of feared crimes, *t* (773) = −3.85, *p* < 0.001; namely, females reported a higher mean value than males (3.41 vs. 2.79, respectively). As can be seen in Table 3, males and females only differed on the perceptions of occurrence of sexual offenses and domestic violence against an intimate partner. In both cases, females perceived the crimes as more frequent than males. There were no gender differences on the total number of perceived crime occurrences; *t* (773) = −1.72, *p* = 0.086. When asked about factors that promote crime, males and females presented similar results. Nonetheless, in comparison to males, females tended to justify crime with juvenile conflicts and delinquency, poor lighting, and reduced presence of people during night. Incapacity to act on the part of police officers and the low severity of punishment were crime factors especially reported by males.

## 4. Discussion

Although traditional perspectives assume college campuses to be safe places [1,2], empirical data on victimization [13,36], in addition to some tragic events (e.g., shootings), suggest that campuses can be a hotspot for crime. Nonetheless, it should be noted that most of the evidence has relied on Anglo-American cultures, making it important to focus on other cultures [35]. Therefore, in this exploratory study, we focused on perceptions of (in)security and crime on a Portuguese higher education campus, analyzing different parameters (e.g., perception of occurrence, most feared crimes) of subjective insecurity [8]. Additionally, we compared males and females on those variables. Despite expected differences, there were several similarities with findings from other countries, which will next be presented and discussed.

As found by Merianos, King, and Vidourek [14], our results suggest that the majority of higher education students felt safe on campus. When we asked students to explain their answers, previous experience/observation was the main presented reason. Indeed, similarly to other international studies [15,46,47], in a previous work [10], we concluded that there was a significant association between perception of (in)security and victimization (both direct and indirect). It should be noted that a slightly higher percentage of students reported feelings of insecurity (26.6%) than those observed by Starkweather [12], namely 20.6%. According to the participants’ points of view, the presence of crime/danger and the urban environmental degradation/space distribution cause this perception. The campus environment is where students spend most of their time: they attend classes, study, live, socialize, and it seems to also be a place where they become victims of crime [10]. Moreover, Porto’s campus layout and design may also be influencing perceptions of crime and fear. For instance, according to the situational crime prevention through environmental design principles theory [28], we hypothesized that the extension of the campus may add difficulty to access control and target hardening, while the presence of some incivilities and urban degradation may foster feelings of unsafety.

A couple of results concerning the (mis)match between the most feared crimes and the perception of occurrence deserve a comment. First, robbery, physical assault, and theft were identified not only as the most feared crimes, but also as the most common on Porto’s campus. This supports previous findings from the international literature [11,13,15], but also national-community-level trends [41]. Moreover, in our previous work [10], victims reported mainly being victims of those crimes, and the media also pay special attention to these kinds of episodes [37,38]. Therefore, we can conclude that there seems to be a match between the most feared crimes and perceptions of occurrence, not only on our study but also when comparing with other indicators.

Domestic violence involving intimates and sexual offenses seem to represent special issues. Sexual offenses, despite a low percentage of participants reporting them as a common crime on campus, were feared by almost one-third. Although students seem to report a higher frequency of sexual assault than non-students [25], results about perception of occurrence seem to be in line with national data provided by the Associação Portuguesa de Apoio à Vítima [48], which concluded that only 435 episodes of sexual assault were reported to the police throughout five years, and those aged between 18 and 24 were not the most vulnerable group (i.e., they represented 26.22% of the victims). Moreover, data from the same source about the spaces of occurrence suggested that, in 2017, public spaces—among which higher education campuses can be included—were less reported by the victims of sexual violence. Moreover, results about the fear of sexual offenses are well documented in the literature [15,22,23,49], and according to Hilinski [50] “those studies that do exist have found that societal, individual, institution, spatial, and temporal factors all contribute to female’s fear of rape and sexual assault” (p.86). Lastly, domestic violence involving intimates presented very low values on fear of crime (7.2%) and perception of occurrence (6.60%); these results differ from both international studies [1,51,52] and national evidence [53]. For instance, according to the Associação Portuguesa de Apoio à Vítima, between 2013 and 2017, 36,528 support incidents were opened, and intimate partners represented 59% of the offenders [54]. Additionally, among those aged 18 and 24, there was an increasing trend of victimization from 2013 to 2017, respectively 17.3% and 18.8%. Domestic violence involving intimates seems to be detrimental not only for physical and psychological health but also to academic performance and even dropout rates [55].

Although there are several studies about the conditions that promote crime and fear on higher education campuses [2,46,56], to our best knowledge this was the first study that asked higher education students about the topic. According to our participants, alcohol/drug consumption, juvenile delinquency, poverty/unemployment, and insufficient policing were the main factors presented. Some of those can be seen as evidence in support of the situational opportunity theories [26] and overlap with lifestyle behaviors and personal characteristics traditionally associated with crime and fear [29]. Moreover, Porto’s campus layout and design may also be relevant. According to the situational crime prevention through environmental design principles [28], we suggested that the permeability of the borders and the extension of the campus may increase the difficulty of access control and target hardening and increase the vulnerability of inhabitants’ social conditions (e.g., unemployment). Lastly, similarly to other countries [5], in Portugal, there is no specific police force to deal with campus criminality or community policing, which can explain our findings. Indeed, according to Patton and Gregory [57], despite there being no differences in the perception of (in)security between those students that attended a campus with a security department and those that attended a campus with a police department, the authors found that “students attending a campus with no security or police department were shown to have the greatest concern of campus safety” (p. 455).

A last comment should be made regarding gender differences on perceptions of (in)security and crime. Overall, females reported higher perceptions of (in)security and a higher number of feared crimes than males. Moreover, females feared not only those crimes usually associated with female victimization (i.e., sexual assault or domestic violence) but also those crimes related to male victimization (e.g., robbery, physical aggression). This is a good agreement with the findings of a meta-analytic study by Collins [35] that found that gender was the strongest predictor of fear of crime—females seem to be twice as likely to self-report fear of crime than males. Additionally, our findings provide further evidence for the shadow of sexual assault hypothesis [22,50]. Nonetheless, according to a study by Sutton and Farrall [24], “men produce a pattern of responses in which fear of crime is inversely related to socially desirable responding” (p. 221), which suggests an unwillingness of males to report insecurity and fear. It should also be noted that variables and factors related to fear in females and males are far from being well understood; indeed, based on Schafer, Huebner, and Bynum’s results [58], predictors seemed to be gendered, and the predictive models (that tested individual factors, fear facilitators, and fear inhibitors) performed better for males than females.

While the current findings have contributed to our understanding of the factors that influence perceptions of (in)security and crime among higher education students, there are some limitations. First, despite being focused on a not previously studied population (i.e., a Portuguese sample), which certainly presented its own cultural specificities, it can be included in the WEIRD societies (i.e., Western, educated, industrialized, rich, and democratic societies) and, consequently, is hard to generalize to other populations [59]. Moreover, this study focused solely on perceptions of (in)security and crime within the context of Porto’s campus and students, based on convenience sampling; as a result, the findings cannot be generalized to other settings or groups. Indeed, attending to space characteristics and specificities, our data are informative about that area but not necessarily about others. Third, the findings and patterns obtained here should be applied to other groups (e.g., professors, faculty, and, in this particular case, also passersby) besides students. Another potential limitation was the application of different methods of data collection, namely a web survey and a paper-and-pencil format. This methodological option was taken up in accordance with the higher education institution’s principals, who defined the more convenient strategy of data collection. Nonetheless, this mixed method of data collection may have impacted the findings, and this potential impact should be studied in future studies. Lastly, the findings may be biased by social desirability because the data were collected through a self-report method. 

Considering the limitations and the strengths of this study—for instance, it adds to the existing literature by examining new variables (e.g., factors that promote crime), asking about fear of specific crimes [60], and using a wording that respondents understood [61]—more research and work needs to be done in several areas. In particular, in line with other studies [11,15], it would be relevant to examine other variables, such as a comparison between day and night or the relationship between targets and offenders. Similarly to the studies developed by authors such Jakobi and Põdör [62] or Fuhrmann, Huynh, and Scholz [63] that related fear of crime maps and official crime statistics, it would be interesting to compare whether those areas where students felt more unsafe match the areas were more criminal incidents happens [62]. Future work should also focus on the impact of the perception of (in)security on several domains (lifestyle, academic performance, interpersonal relationships, etc.) as well as the behaviors and other measures adopted by higher education students to deal with it. Additionally, it would also be crucial to assess the physical spaces (e.g., incivilities) and to characterize the built environment, for instance through crime prevention through environmental design. Lastly, institutional factors, social control, and policing are topics that should be further studied. For instance, according to Jacobsen [2], the number of security measures that were adopted by higher education institutions, the female to male enrolment ratio, and the geographic size of campuses were predictors of violent crime. 

## 5. Conclusions

Briefly, this study enlightened that robbery, theft, and public property damage were perceived as common crimes on campus and more than 70% of the students considered crime on campus to be increasing, mainly due to observation/experience. A model was developed to predict (in)security, including sociodemographic and criminal variables, which explained nearly 90% of the cases. Female students seemed to be particularly at risk of feeling unsafe. Additionally, female students seemed to be particularly afraid of robbery, physical assault, sexual violence, and domestic violence, while males feared mainly thefts. The findings from the current study have several practical implications for campus security. Indeed, as Boateng and Adjekum-Boateng claimed [20]:

*“knowing about how students’ fear of crime develops will help in making policies that target the underlying causes of students’ fear. These types of policies have a greater probability of achieving their intended outcomes than policies developed based on general assumptions about the public. In addressing the underlying causes of fear of crime, there is no one-size-fits-all solution; every group requires specific sets of policies tailored to its own needs.”*

(p. 153)

Based on Merianos, King, and Vidourek’s study about factors that promote the perception of safety, students evaluated as particularly useful the presence of police officers, professors, faculty, and other students on campus [14]. Environmental conditions, knowledge about contact information in case of emergency, and to be e-mailed about crimes on and around campus were also valued by students. Moreover, lighting, communication about safety, and crime-related services were identified as issues needing improvement. To our best knowledge, none of these measures have been assessed or applied in a Portuguese case; consequently, they should be the focus of careful analysis, discussion, prioritization, and implementation for this particular community [34,62]. This was a descriptive exploratory study; therefore, it seems to be reasonable to establish a work group or task force involving students, police, administrators, faculty, professors, parents, politicians, and researchers in an effort to further analyze and prevent perceptions of insecurity and crime on campus. Indeed, both social and physical changes, at community and individual levels, should be designed and applied in order to create a safer learning environment [13,14]. Additionally, the development of awareness, educational, and prevention campaigns or programs may also be based on our findings. These campaigns or programs may be focused on specific targets (e.g., male victims) and topics (e.g., domestic violence, victim rights). Moreover, measures and policies implemented to decrease crime occurrences and increase security perceptions should be carefully followed and their efficacy should be evaluated. Therefore, the different stakeholders involved in campus safety should consider the promotion of safety policies and practices [1].

## Figures and Tables

**Table 1:** Participants Description (*N* = 775).

Variables	Frequency	
	*n*	%
Gender		
Male	356	45.9
Female	419	54.1
Nationality		
Portuguese	731	94.3
Other	44	5.7
Brazilian	11	28.2
Spanish	10	25.6
French	7	17.9
Other	11	28.4
Marital status		
Single	727	9.38
Married/cohabiting	41	5.3
Divorced/separated	5	0.6
Other	2	0.3
Education		
Undergraduate	629	81.1
Postgraduate	124	8.1
Doctoral studies	8	1.0
Other	13	1.7
Attendance status		
Full time	658	84.9
Part time	117	15.1
Years of attendance		
3 or less years	593	76.5
4 to 6 years	151	19.5
7 to 9 years	22	2.8
10 or more years	9	1.2
University domain		
Engineering	316	40.8
Sports	30	3.9
Health sciences	24	3.1
Education and psychology	23	3.0
Economics and management	13	1.7
Mixed	352	45.4
Other	17	2.2

**Table 2:** Frequencies of Most Feared Crimes and Perception of Occurrence by Type of Crime.

Type of Crime	Most Feared Crimes % (*n*)	Perception of Occurrence % (*n*)
Robbery	67.9 (526)	43.90 (340)
Theft	44.4 (344)	40.90 (317)
Public property damage	12.5 (97)	39.00 (302)
Physical assault	55.0 (426)	29.20 (226)
Drug trafficking	19.2 (149)	28.60 (222)
Road traffic crime	18.7 (145)	16.80 (130)
Fraud	12.9 (100)	11.70 (91)
Sexual offense	31.6 (245)	11.70 (91)
Burglary		
Residence	23.0 (178)	8.40 (65)
Commercial property	4.6 (36)	7.60 (59)
Domestic violence		
Intimates	7.2 (56)	6.60 (51)
Children	7.6 (59)	2.30 (18)
Elderly	7.7 (60)	2.10 (16)
Arms traffic	15.4 (119)	1.20 (9)

**Table 3:** Results of Binary Regression Model to Predict Perception of Insecurity.

Individual Predictors	B	Wald (1)	*p*
Step 1	−1.26	28.03	
Gender	0.25	13.50	<0.001
Age	1.08	2.94	<0.001
Nationality	0.50	0.97	0.086
Marital status	0.45	2.55	0.326
Education	−0.10	0.96	0.110
Status attendance	−1.51	37.76	0.757
Years of attendance			<0.001
University domain			
Dummy 1	−0.57	4.09	0.043
Dummy 2	−2.18	0.22	0.638
Step 2			
Gender	−2.48	32.97	<0.001
Age	0.53	22.55	<0.001
Nationality	−0.30	0.08	0.785
Marital status	0.35	0.25	0.619
Education	1.78	13.92	<0.001
Status attendance	−0.26	0.29	0.591
Years of attendance	−1.78	20.23	<0.001
University domain			
Dummy 1	−0.24	0.34	0.560
Dummy 2	1.35	2.95	0.086
Perceived trend of crime	2.22	41.86	<0.001
Number of feared crimes	0.14	3.71	0.054
Number of crime occurrences	0.37	29.22	<0.001
Number of crime promoters	0.84	65.90	<0.001

**Note:** Variable to predict: perception of insecurity (1 = unsafe; 0 = safe); categorical variables: gender (1 = male; female = 0); nationality (Portuguese = 1; other = 0); marital status (1 = single; 0 = other); education (1 = graduated degree; 0 = other), status of attendance (1 = 3 or less years; 0 = 4 or more years); university domain (Dummy 1: engineering = 1; other = 0; Dummy 2: mixed = 1, specific = 0); perceived trend of crime (increasing = 1; not increasing = 0).

**Table 4:** Frequency and Chi-Square Tests for Feared Crimes, Perception of Occurrence, and Crime Promoter Factors by Gender.

Variables	Gender		*Χ*^2^(1)	*p*
	Female (*N* = 419) % (*n*)	Male (*N* = 356) % (*n*)		
Feared crimes				
Robbery	38.7 (300)	29.2 (226)	5.81	0.016
Theft	21.0 (163)	23.4 (181)	11.12	0.001
Public property damage	5.8 (45)	6.7 (52)	2.63	0.105
Physical assault	31.6 (245)	23.4 (181)	4.53	0.033
Drug trafficking	9.5 (74)	9.7 (75)	1.44	0.230
Road traffic crime	10.7 (83)	8.0 (62)	0.73	0.395
Fraud	7.6 (59)	5.3 (41)	1.23	0.289
Sexual offense	27.9 (216)	3.7 (29)	167.73	0.001
Burglary—residence	13.3 (103)	9.7 (75)	1.34	0.246
Burglary—commercial property	2.6 (20)	2.1 (16)	0.03	0.854
Domestic violence—intimates	5.2 (40)	2.1 (16)	7.33	0.007
Domestic violence—children	5.4 (42)	2.2 (17)	7.54	0.006
Domestic violence—elderly	5.0 (39)	2.7 (21)	3.13	0.077
Arms traffic	7.5 (58)	7.9 (61)	1.61	0.205
Perception of occurrence				
Robbery	25.4 (197)	18.5 (143)	3.67	0.056
Theft	20.9 (162)	20.0 (155)	1.89	0.169
Public property damage	21.2 (164)	17.8 (138)	0.01	0.920
Physical assault	15.9 (123)	13.3 (103)	0.02	0.897
Drug trafficking	14.5 (112)	14.2 (110)	1.64	0.201
Road traffic crime	10.2 (79)	6.6 (51)	2.83	0.093
Fraud	6.8 (53)	4.8 (38)	0.72	0.395
Sexual offense	8.9 (69)	2.8 (22)	19.66	0.001
Burglary—residence	4.9 (38)	3.5 (27)	0.552	0.457
Burglary—commercial property	4.6 (36)	3.0 (23)	1.24	0.265
Domestic violence—intimates	4.8 (37)	1.8 (14)	7.51	0.006
Domestic violence—children	1.7 (13)	0.6 (5)	2.45	0.118
Domestic violence—elderly	1.4 (11)	0.6 (5)	1.42	0.234
Arms traffic	0.6 (5)	0.5 (4)	0.01	0.928
Factors that promote crime				
Alcohol/drug consumption	29.7 (230)	23.5 (182)	1.10	0.295
Poverty/unemployment	23.0 (178)	23.1 (179)	4.71	0.030
Family problems	7.9 (61)	8.3 (64)	1.66	0.197
Juvenile conflicts and delinquency	24.6 (191)	24.1 (187)	3.71	0.054
Poor lighting	17.8 (138)	9.5 (74)	14.30	0.001
Poor accessibilities	9.0 (70)	5.5 (43)	3.31	0.069
Lack of green spaces	3.1 (24)	3.4 (26)	0.79	0.374
Presence of strangers	15.0 (116)	12.0 (93)	0.24	0.625
Reduced presence of people during night	23.4 (181)	16.4 (127)	4.55	0.033
Insufficient policing	23.2 (180)	19.1 (148)	0.15	0.697
Incapacity to act on the part of police officers	8.8 (68)	10.3 (80)	4.86	0.028
Low severity of punishment	11.2 (87)	13.0 (101)	6.06	0.014

## Data Availability

Dataset and syntaxes are available upon request to the authors.

## Supplementary Materials

The following supporting information can be downloaded at: https://www.mdpi.com/article/10.3390/ejihpe12020015/s1.
