# Supplementary material for: Is Campus a Place of (In)Security and Crime? Perceptions and Predictors among Higher Education Students
Source: Eur J Investig Health Psychol Educ. 2022 Feb 2;12(2):193–208. doi: 10.3390/ejihpe12020015 (PMC8871454; doi:10.3390/ejihpe12020015)
Supplement: Supplementary file 1 [file ejihpe-12-00015-s001.zip › ejihpe-1502867-supplementary.pdf]

(In)Segurança e Criminalidade

Universidade Fernando Pessoa
(Ana Sani & Laura Nunes)

* Required

O inquérito a que vai responder visa recolher informação para a realização de um estudo sobre a perceção de segurança/insegurança e a criminalidade na área onde estuda atualmente.

A sua participação será anónima e confidencial, destinando-se, única e exclusivamente, à realização do estudo cujos resultados ajudarão a melhorar as condições de segurança da área onde estuda atualmente (Polo Universitário de Asprela).

O inquérito divide-se em 5 partes: a parte A prende-se com dados sociodemográficos; a parte B procura apurar a forma como sente a segurança na sua área de residência; a parte C questiona sobre experiências de vitimação; a parte D tenta avaliar como operar o controlo social no lidar com o fenómeno criminal e, por fim, a parte E pretende apreciar o envolvimento dos cidadãos na resolução dos problemas da comunidade em que estuda atualmente. O preenchimento do questionário demora, aproximadamente, 30 minutos.

Agradece-se a sua participação consciente e empenhada.

Declaração de Consentimento Informado

Declaro que aceito participar no estudo intitulado Diagnóstico Local de Segurança do Polo Universitário de Asprela, de que são responsáveis Ana Isabel Sani e Laura M. Nunes, investigadoras e docentes na Universidade Fernando Pessoa.

Declaro que, antes de optar pela minha participação, tomei conhecimento dos objetivos do estudo, de todos os aspetos que considerei importantes para a minha decisão e do que tenho de fazer para participar. Fui também informado(a) da duração esperada e dos procedimentos do estudo, tendo-me sido dadas garantias de anonimato e de confidencialidade, além de que me foi transmitido o direito que me assiste de recusar participar ou de cessar a minha participação, em qualquer momento, sem quaisquer consequências para mim.

Tendo compreendido todas as informações que me foram dadas a respeito, aceito participar voluntariamente, colaborando com total sinceridade.

1. *

Check all that apply.

☐ Concordo e participo voluntariamente

Parte A: Dados Sociodemográficos

2. **1.1. Sexo** *

Mark only one oval.

◯ Masculino

◯ Feminino

3. **1.2. Idade** *

____

4. **1.3. Nacionalidade** *

Mark only one oval.

- Portuguesa
- Estrangeira

5. **1.3.b Qual?**

(se estrangeira)

6. **1.4. Estado Civil** *

Mark only one oval.

- Solteiro(a)
- Casado(a) / União de Facto
- Divorciado(a) / Separado(a)
- Viúvo(a)
- Outro

7. **1.4.b Qual?**

(se outro)

8. **1.5. Escolaridade** *

Mark only one oval.

- Primeiro Ciclo (Licenciatura) – 1º Ano
- Primeiro Ciclo (Licenciatura) – 2º Ano
- Primeiro Ciclo (Licenciatura) – 3º Ano
- Segundo Ciclo (Mestrado) – 1º Ano
- Segundo Ciclo (Mestrado) – 2º Ano
- Terceiro Ciclo (Doutoramento)
- Outro

9. **1.5.b Qual?**

(se outro)

10. **1.5.c Estabelecimento de Ensino Superior que frequenta** *

11. **1.6.a Rua de residência no Porto**

12. **1.6. Tipo Habitação** *
Mark only one oval.

- Apartamento
- Casa
- Outro

13. **1.7. Situação Ocupacional** *
Mark only one oval.

- Estudante
- Trabalhador-Estudante

14. **1.7.b Área laboral**
(se trabalhador-estudante)

15. **1.8. Com quem vive?** *
Mark only one oval.

- Família nuclear (e.g., Cônjuge/companheiro e filhos; Pais; Pais e irmão(s))
- Família nuclear alargada (e.g., Avó(s) e irmão(s); Pais e irmão(s) e sobrinho(s); Pais e avó(s); Cônjuge e filho(s) e Mãe; Cônjuge e filho(s), netos e genro; Pais e irmão(s) e filho(s); Filho(s) e irmão(s))
- Família alargada (e.g., tios e primos; avós; irmã cunhados e sobrinhos)
- Pares (e.g., universitários; institucionalizados)
- Não se aplica (Se vive só)

Parte B: Perceção de Segurança/Insegurança

16. **2.1. Refira se considera que estuda numa área segura** *
Mark only one oval.

- Sim
- Não

17. **2.1.a Explique porquê** *

18. **2.2. Diga se lhe parece que a criminalidade tem aumentado, na área onde que estuda** *
Mark only one oval.

- Sim
- Não

19. **2.2.a Explique porquê** *

2.3. Da lista seguinte, atendendo ao seu ponto de vista, assinale os crimes que mais frequentemente ocorrem na área onde estuda atualmente

(Escollha uma ou mais opções)

20. **2.3.a.**
Check all that apply.
- [ ] Burla

21. **2.3.b.**
Check all that apply.
- [ ] Furto (ex: automóvel)

22. **2.3.c**
Check all that apply.
- [ ] Roubo (ex: esticão)

23. **2.3.d.**
Check all that apply.
- [ ] Assalto a residência

24. **2.3.e.**
Check all that apply.
- [ ] Assalto a estabelecimento comercial

25. **2.3.f.**
Check all that apply.
- [ ] Ofensa sexual

26. **2.3.g.**
Check all that apply.
- [ ] Agressão física

27. **2.3.h.**
Check all that apply.
- [ ] Violência doméstica: contra/entre menores

28. **2.3.i.**
Check all that apply.

- [ ] Violência doméstica: contra/entre namorados/cônjuges,

29. **2.3.j.**
Check all that apply.

- [ ] Violência doméstica: contra/entre idosos

30. **2.3.k.**
Check all that apply.

- [ ] Danos a espaços/equipamentos públicos

31. **2.3.l.**
Check all that apply.

- [ ] Crimes rodoviários

32. **2.3.m.**
Check all that apply.

- [ ] Tráfico de drogas

33. **2.3.n.**
Check all that apply.

- [ ] Tráfico de armas

34. **2.3.o.**
Check all that apply.

- [ ] Desconhece/Nenhum

35. **2.3.p.**
Check all that apply.

- [ ] Outros

36. **2.3.pl Quais?**
(se indicou outros)

____
____
____
____
____

2.4. Da lista seguinte, atendendo ao seu ponto de vista, assinale os crimes que mais teme na área onde estuda atualmente

(escolha uma ou mais opções)

37. **2.4.a.**
Check all that apply.

- [ ] Burla

38. **2.4.b.**
Check all that apply.

- [ ] Furto (ex: automóvel)

39. **2.4.c.**
Check all that apply.

- [ ] Roubo (ex: esticão)

40. **2.4.d.**
Check all that apply.

- [ ] Assalto a residência

41. **2.4.e.**
Check all that apply.

- [ ] Assalto a estabelecimento comercial

42. **2.4.f.**
Check all that apply.

- [ ] Ofensa sexual

43. **2.4.g.**
Check all that apply.

- [ ] Agressão física

44. **2.4.h.**
Check all that apply.

- [ ] Violência doméstica: contra/entre menores

45. **2.4.i.**
Check all that apply.

- [ ] Violência doméstica: contra/entre cônjuges/namorados

46. **2.4.j.**
Check all that apply.

- [ ] Violência doméstica: contra/entre idosos

47. **2.4.k.**
Check all that apply.

- [ ] Danos a espaços/equipamentos públicos

48. **2.4.l.**
Check all that apply.

- [ ] Crimes rodoviários

49. **2.4.m.**
Check all that apply.

- [ ] Tráfico de drogas

50. **2.4.n.**
Check all that apply.

- [ ] Tráfico de armas

51. **2.4.o.**
Check all that apply.

- [ ] Desconhece/Nenhum

52. **2.4.p.**
Check all that apply.

- [ ] Outros

53. **2.4.pl Quais?**
(se indicou outros)

2.5. Da lista seguinte, atendendo ao seu ponto de vista, assinale as condições que, na área onde estuda atualmente, mais lhe parecem favorecer a ocorrência de crime

(escolha uma ou mais opções)

54. **2.5.a.**
Check all that apply.

- [ ] Consumo de drogas/álcool

55. **2.5.b.**
Check all that apply.

- [ ] Pobreza/Desemprego

56. **2.5.c.**
Check all that apply.

- [ ] Problemas familiares

57. **2.5.d.**
Check all that apply.

- [ ] Conflitos e delinquência juvenil

58. **2.5.e.**
Check all that apply.

- [ ] Má iluminação pública

59. **2.5.f.**
Check all that apply.

- [ ] Maus acessos/arruamentos

60. **2.5.g.**
Check all that apply.

- [ ] Ausência de espaços verdes/de lazer

61. **2.5.h.**
Check all that apply.

- [ ] Presença de pessoas estranhas

62. **2.5.i.**
Check all that apply.

- [ ] Reduzido movimento durante a noite

63. **2.5.j.**
Check all that apply.

- [ ] Policiamento deficitário

64. **2.5.k.**
Check all that apply.

- [ ] Incapacidade de atuação dos agentes de autoridade

65. **2.5.l.**
Check all that apply.

- [ ] Pouca severidade para com os ofensores

66. **2.5.m.**
Check all that apply.

- [ ] Desconhece/Nenhum

67. **2.5.n**
Check all that apply.

- [ ] Outros

68. **2.5.nI Quais?**
(se indicou outros)

2.6. Da lista seguinte, atendendo ao seu ponto de vista, assinale as incivilidades que mais frequentemente se verificam na área onde estuda atualmente

(escolha uma ou mais opções)

69. **2.6.a.**
Check all that apply.

- [ ] Urinar na via pública

70. **2.6.b.**
Check all that apply.

- [ ] Produzir ruído na via pública

71. **2.6.c.**
Check all that apply.

- [ ] Deixar as fezes de animais de companhia na via pública

72. **2.6.d.**
Check all that apply.

- [ ] Dispersar lixo pela rua

73. **2.6.e.**
Check all that apply.

- [ ] Violar regras de trânsito

74. **2.6.f.**
Check all that apply.

- [ ] Estacionar de forma caótica

75. **2.6.g.**
Check all that apply.

- [ ] Peditórios ilegais (ex. arrumadores de automóveis)

76. **2.6.h.**
Check all that apply.

- [ ] Desconhece/Nenhum

77. **2.6.i.**
Check all that apply.

- [ ] Outros

78. **2.6.il Quais?**
(se indicou outros)

Parte C: Vitimação

79. **3.1. Diga se, nos últimos 5 anos, foi vítima de crime na área onde estuda atualmente** *
(se foi vítima de mais de 1 crime, refira-se ao mais grave)
Mark only one oval.

- ( ) Sim *Skip to question 80.*
- ( ) Não *Skip to question 95.*

Reportando-se aos últimos 5 anos, referiu ter sido vítima de crime na área onde estuda atualmente.

80. **3.1.1. Qual?** *

81. **3.1.2. Descreva os danos sofridos** *
(físicos/psicológicos/materiais)

Relativamente ao crime que acaba de descrever, responda às questões seguintes:

82. **3.1.3. Em que altura ocorreu:** *
Mark only one oval.

- De dia
- De noite
- Não Sabe

83. **3.1.4. Onde se deu a ocorrência:** *
Mark only one oval.

- Em Casa
- Na Rua
- Outro

84. **3.1.4.b Qual?**
(se indicou outro)

85. **3.1.5. Nessa altura encontrava-se:** *
Mark only one oval.

- Sozinho
- Acompanhado
- Não Sabe

86. **3.1.6. O ofensor era alguém:** *
Mark only one oval.

- Conhecido
- Estranho
- Não Sabe

87. **3.1.7. Diga se contactou as autoridades** *
Mark only one oval.

- Não
- Sim

88. **3.1.7.a Explique porquê** *
(de acordo com a sua resposta prévia, quer tenha indicado sim ou não)
Mark only one oval.

- Não confia
- Não vale a pena
- Falta de tempo
- Medo
- Confia
- Vale a pena
- Seguradora
- Outro

89. **3.1.7.b Qual?**
(se indicou outro)

90. **3.1.8. Diga se formalizou oficialmente a queixa** *
Mark only one oval.

- Não
- Sim

91. **3.1.8.a Explique porquê** *

92. **3.1.9. Refira as medidas tomadas pelas autoridades** *

93. **3.1.10. Diga se ficou satisfeito com essas medidas** *
Mark only one oval.

- Não
- Sim

94. **3.1.10.a Explique porquê** *

95. **3.2. Refira se, nos últimos 5 anos, alguém seu conhecido ou colega foi vítima de crime na área onde estuda atualmente** *
(se foi vítima de mais de 1 crime, refira-se ao mais grave).
Mark only one oval.

- Sim *Skip to question 96.*
- Não *Skip to question 112.*

Reportando-se aos últimos 5 anos, referiu que alguém seu conhecido ou colega foi vítima de crime na área onde estuda atualmente

96. **3.2.a Quem?** *

97. **3.2.1. Que crime** *

98. **3.2.2. Descreva os danos sofridos:** *
(físicos/psicológicos/materiais)

Relativamente ao crime que acaba de descrever, responda às questões seguintes:

99. **3.2.3. Em que altura ocorreu:** *
Mark only one oval.

- De dia
- De noite
- Não sabe

100. **3.2.4. Onde se deu a ocorrência:** *

Mark only one oval.

- Em Casa
- Na Rua
- Não Sabe
- Outro local

101. **3.2.4.b Qual?**

(se indicou outro local)

102. **3.2.5. Nessa altura esse conhecido ou colega encontrava-se:** *

Mark only one oval.

- Sozinho
- Acompanhado
- Não sabe

103. **3.2.6. O ofensor era alguém:** *

Mark only one oval.

- Conhecido
- Estranho
- Não sabe

104. **3.2.7. Diga se o seu conhecido ou colega contactou as autoridades** *

Mark only one oval.

- Não
- Sim
- Não sabe

105. **3.2.7.a Explique porquê** *

(de acordo com a sua resposta, se indicou sim ou não)

Mark only one oval.

- Não confia
- Não vale a pena
- Falta de tempo
- Medo
- Confia
- Vale a pena
- Seguradora
- Não sabe
- Outro

106. **3.2.7.b Qual?**
(se indicou outro)

107. **3.2.8. Diga se o seu conhecido ou colega formalizou oficialmente a queixa** *
Mark only one oval.

- Não *After the last question in this section, skip to question 112.*
- Sim
- Não Sabe *After the last question in this section, skip to question 112.*

108. **3.2.8.a Explique porquê**

Referiu que o seu conhecido ou colega formalizou oficialmente a queixa.

109. **3.2.9. Refira as medidas tomadas pelas autoridades**

110. **3.2.10. Diga se o seu conhecido ou colega ficou satisfeito com essas medidas** *
Mark only one oval.

- Não
- Sim
- Não sabe

111. **3.2.10.a Explique porquê**

Parte D: Controlo Social

112. **4.1. Refira se considera que os agentes de segurança fazem tudo para garantir a segurança na área onde estuda atualmente** *
(escolha apenas uma opção de resposta)
Mark only one oval.

- Sempre
- Quase Sempre
- Quase Nunca
- Nunca
- Não Sabe

113. **4.1.1. Explique a sua opção de resposta** *

114. **4.2. Indique o grau de satisfação em relação à atuação dos agentes de segurança, na área onde estuda atualmente** *
(escolha apenas uma opção de resposta)
Mark only one oval.

- Muito Satisfeito(a)
- Satisfeito(a)
- Pouco Satisfeito(a)
- Nada Satisfeito(a)
- Não Sabe

115. **4.2.1. Explique a sua opção de resposta** *

116. **4.3. Refira com que frequência recorre à ajuda de colegas, amigos ou vizinhos, em caso de ter algum problema, na área onde estuda atualmente** *
(escolha apenas uma opção de resposta)
Mark only one oval.

- Sempre
- Quase Sempre
- Quase Nunca
- Nunca

117. **4.3.1. Explique a sua opção de resposta** *

118. **4.4. Refira com que frequência recorre à ajuda das entidades de apoio na área onde estuda atualmente, em caso de ter algum problema** *
(escolha apenas uma opção de resposta)
Mark only one oval.

- Sempre
- Quase Sempre
- Quase Nunca
- Nunca

119. **4.4.1. Explique a sua opção de resposta** *

120. **4.4.2. Se recorre ao apoio de entidades, mencione quais:**

Parte E: Participação Comunitária

121. **5.1. Diga há quantos anos estuda nesta área (Polo Universitário de Asprela)** *
Mark only one oval.

- 3 Anos ou menos
- 4 a 6 Anos
- 7 a 9 Anos
- 10 Anos ou mais

5.2. Refira o que gostaria de ver melhorado na área onde estuda atualmente, no sentido de que tivesse mais qualidade de vida

(Faça uma lista por ordem de importância, colocando no espaço 1 a medida que lhe parece mais importante e prosseguindo até ao espaço 5, onde colocará a medida que lhe parece menos importante)

122. **5.2.1**
(em primeiro lugar)

123. **5.2.2**
(em segundo lugar)

124. **5.2.3**
(em terceiro lugar)

125. **5.2.4**
(em quarto lugar)

126. **5.2.5**
(em quinto lugar)

5.3. Refira o que gostaria de ver melhorado na área onde estuda atualmente, no sentido de que houvesse mais segurança

(Faça uma lista por ordem de importância, colocando no espaço 1 a medida que lhe parece mais importante e prosseguindo até ao espaço 5, onde colocará a medida que lhe parece menos importante)

127. **5.3.1**
(em primeiro lugar)

128. **5.3.2**
(em segundo lugar)

129. **5.3.3**
(em terceiro lugar)

130. **5.3.4.**
(em quarto lugar)

131. **5.3.5.**
(em quinto lugar)

132. **5.4. Diga se estaria disposto a colaborar no sentido de que houvesse mais segurança na área onde estuda atualmente** *
(escolha apenas uma opção de resposta)
Mark only one oval.

- Sempre
- Quase Sempre
- Quase Nunca
- Nunca

133. **5.4.a. Se respondeu sempre ou quase sempre, especifique de que forma**

134. **5.4.b. Se respondeu quase nunca ou nunca, explique porquê**

135. **5.5. Diga qual a força da sua ligação ao local onde estuda atualmente** *
(escolha apenas uma opção de resposta)
Mark only one oval.

- Muito forte
- Forte
- Pouco Forte
- Nada Forte

136. **5.5.1. Explique a sua opção de resposta** *

Muito Obrigado pela sua Colaboração!

Powered by
Google Forms
